# Glaucoma management in Sweden – results from a nationwide survey

**DOI:** 10.1111/j.1755-3768.2011.02273.x

**Published:** 2013-02

**Authors:** Christina Lindén, Boel Bengtsson, Albert Alm, Berit Calissendorff, Ingemar Eckerlund, Anders Heijl

**Affiliations:** 1 Department of Clinical Sciences, Ophthalmology, Umeå UniversityUmeå, Sweden; 2 Department of Clinical Sciences Malmö, Ophthalmology, Skåne University Hospital, Lund UniversityMalmö, Sweden; 3 Department of Neurosciences, Ophthalmology, Uppsala UniversityUppsala, Sweden; 4 Department of Clinical Neuroscience, Ophthalmology, St. Erik Eye HospitalStockholm, Sweden; 5 SBU – The Swedish Council on Health Technology AssessmentStockholm, Sweden

**Keywords:** equipment, fundus photograph, glaucoma, management, visits, visual field

## Abstract

**Purpose:**

To report the results from a nationwide survey on glaucoma management in Sweden, performed as a part of an Open Angle Glaucoma project conducted by the Swedish Council on Health Technology Assessment 2004–2008.

**Methods:**

In 2005, a survey was distributed to all providers of glaucoma care in Sweden: public eye departments, public outpatient departments and private practices. The questionnaire included questions on number of examined patients, types of examinations during one defined week, internal organization and access to diagnostic equipment. The questionnaire was endorsed by the Swedish Ophthalmological Society. Reminders were sent out to nonresponders.

**Results:**

Response rate was high; 97% (33/34) of eye departments, 85% (39/46) of outpatient departments and 55% (69/125) of private practices. Out of 29 282 visits in ophthalmic care during the study week, 7737 (26%) were related to glaucoma. Diagnostic equipment was generally available; all public eye facilities and 92% of private practices had at least one computerized perimeter, while equipment for fundus photography/imaging was available at 100% of eye departments, 82% of outpatient departments and 62% of private practices. The number of visual field tests and fundus images was rather low. Survey results indicate that patients on the average underwent bilateral field testing every 2nd year and fundus imaging every 8th year.

**Conclusion:**

Glaucoma care generated about a quarter of all patient visits in Swedish ophthalmic care. Access to diagnostic facilities was good. To meet modern standards of glaucoma care, glaucoma damage must be measured and followed more closely than at the time of the survey.

## Introduction

Irrespective of discipline, the vast majority of health care in Sweden is subject to government funding and part of the public health care system. Furthermore, patients with suspect or manifest glaucoma in Sweden are followed by ophthalmologists and ophthalmic nurses, and not by general practitioners or optometrists.

It is generally believed that glaucoma management in Sweden mainly follows national guidelines (Heijl 1997) and a ‘State of the art’ document (Heijl et al. 1998) that were established in 1995 and 1997, respectively, in a collaboration between the Swedish Glaucoma Society, the Swedish Ophthalmological Society and the National Board of Health and Welfare. However, the statistics from Swedish pharmacy sales show considerable variations across the country in patterns of prescription of glaucoma drugs. Official statistics regarding frequency of laser trabeculoplasty and trabeculectomies also show large variations between different areas (Swedish Ophthalmological Society 2007). These facts suggest that the patterns of practice in Sweden need further investigation.

A nationwide survey regarding glaucoma care in Sweden was performed in conjunction with a systematic literature review, concerning the evidence base for diagnostic procedures and treatment of glaucoma. It was initiated by SBU – the Swedish Council on Health Technology Assessment (SBU 2008). The aim was to assess the patterns of diagnostic procedures and follow-up. Special attention was given to detect differences between geographical and/or administrative areas and between academic, public and private health services.

The aim of this paper is to report and discuss the results from this survey.

## Material and Methods

In the autumn of 2005, more than 200 questionnaires were sent to the heads of all public and private eye departments and practices listed in the official list of members of the Swedish Ophthalmological Society (Swedish Ophthalmological Society 2006). The investigation was conducted in cooperation with SBU, and the Swedish Ophthalmological Society. There was no financial incentive to answer, but three reminders and telephone calls promoted the response rate. Confidentiality of response was retained.

An important part of the questionnaire aimed at providing information about internal organization, available equipment, number of examinations, staff and other resources during one specified week, week 42. Health care providers were divided into one of three categories with respect to unit characteristics and in accordance with the official inpatient and outpatient care classification used in the annual report statistics from the Swedish Ophthalmological Society (Swedish Ophthalmological Society 2007). Data on the population broken down by region and age were also retrieved from that report. ‘Public eye departments’ included all public ophthalmic departments, with inpatient care, also academic ones. ‘Public outpatient departments’ were all other public ophthalmology national health care, i.e., units with outpatient care only. ‘Private practices’ were defined as all private ophthalmology health care units irrespective of size of the unit, or whether inpatient or outpatient care. As the vast majority of health care in Sweden is government-funded, private clinics are small, with few exceptions.

Questions included:

1. Does your department/practice have a designated person (physician/nurse) with over-riding responsibility for glaucoma care?
2. Total number of patient visits to physicians and/or nurses during week 42?
3. Number of visits related to glaucoma during the same week?
4. Number of visits to physicians or physicians and nurses?
5. Number of visits to nurses without an appointment with a physician?
6. Number of visual field tests (eyes)? How many with manual perimetry?
7. Number of eyes subjected to fundus photography or computerized imaging?
8. Available diagnostic equipment (answers given by checking a table).

**Figure 1:**
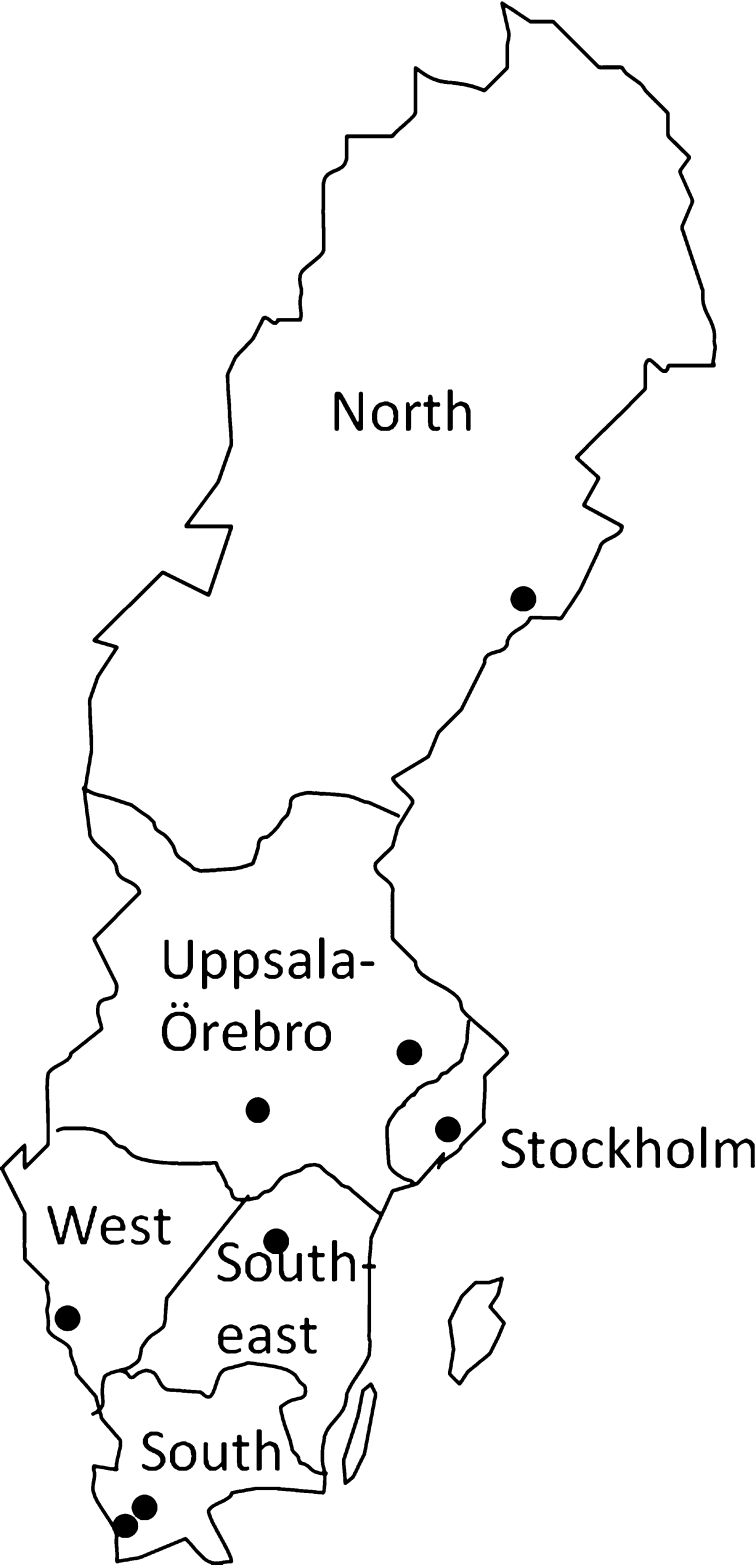
Map of the six national health care regions in Sweden. Cities with university departments are shown.

Answers were analysed based on type of health care providers and also with respect geographical region, i.e. the six national health care regions in Sweden: Northern Region, Uppsala-Örebro Region, Stockholm Region, Western Region, South-east Region or Southern Region (Fig. 1).

## Results

### Visits

The response rate was high. The questionnaire was answered by heads or deputy heads of 33 of 34 (97%) public eye departments, 39 of 46 (85%) public outpatient departments and 69 of 125 (55%) private clinics. Thus, the overall response rate was 90% for public ophthalmic care and 69% when the private clinics were included.

Sixty-five per cent of public eye care providers (86% of the departments, 49% of the outpatient departments) had a designated person with over-riding responsibility for glaucoma care.

During the study week, 29 282 visits in ophthalmological health care in Sweden were reported, and 7737 of these (26%) were related to glaucoma. The proportion of glaucoma visits of all visits was 22%, 30% and 35% for public eye departments, public outpatient departments and private practices, respectively. Seventy-one per cent of all glaucoma visits were taken care of in the public health care, and 29% in private clinics. From these figures, the number of glaucoma-related visits per year in Sweden can be estimated to approximately 325 000.

In public health care, 47% of the visits were appointments with a physician (with or without assistance of a nurse), while ophthalmic nurses handled the remaining 53%. In private care, 86% of the patients met a physician.

### Equipment

All, but one respondent, reported having access to at least one perimeter. All public eye departments and outpatient departments and 92% of the private practices had automated computerized perimeters. Goldmann perimeters were available in 100%, 92% and 56% of the facilities, respectively. The most widely available automated visual field instrument in public departments and clinics was the Humphrey Field Analyzer, reported by 70%. In private practices, it was the high-pass resolution perimeter, 36% had one.

Access to traditional or digital equipment for fundus photography was reported by 100%, 82% and 62% of public departments, outpatient departments and private practices, respectively. Additional imaging instruments for optic nerve head or nerve fibre layer examination were distributed as follows; 13 public departments, one public and one private practice, i.e. 41%, 3% and 2% of the health care facilities, were equipped for optical coherence tomography; four university departments (i.e. 13% of public departments) had both a Heidelberg Retinal Tomograph (HRT) and a GDx Nerve Fiber Analyzer. Furthermore, two private practices (3%) had a HRT instrument.

### Examinations

During the selected study week, visual field testing was performed on between 2779 and 3560 eyes. The range reflects an uncertainty owing to the fact that there was some misunderstanding of the initial instructions and that in those cases, we do not know whether one or both eyes of each patient were examined. Eighty-seven per cent of the visual fields were performed with automated computerized perimetry. The corresponding numbers of photographs or digital fundus images were 739–944. Number of examinations per visit, as well as regional differences in number of visits, glaucoma visits and examinations, is presented in Table 1.

**Table 1:** Number of ophthalmology health care visits, glaucoma-related visits, perimetries and photographs in Sweden during a defined week.

Part of Sweden	Visits, *n*	Glaucoma visits, *n* (% of all visits)	Glaucoma-related perimetries, maximum *n* (% automated)	Glaucoma-related photographs, maximum *n*	Eyes undergoing perimetry/glaucoma visit, *n*	Eyes undergoing photograph/glaucoma visit, *n*
North	3371	934 (28)	387 (93)	110	0.41	0.12
Uppsala-Örebro	5322	1514 (28)	587 (92)	195	0.39	0.13
Stockholm	5304	1793 (34)	913 (83)	190	0.51	0.11
West	5124	1356 (26)	591 (88)	106	0.44	0.08
South-east	3936	1057 (27)	333 (78)	204	0.32	0.19
South	6225	1083 (17)	749 (89)	139	0.69	0.13
Total Sweden	29 282	7737 (26)	3560 (87)	944	0.46	0.12
Academic departments separated from total	5215	979 (19)	344 (87)	85	0.35	0.09

With 3560 visual field tests and assuming that both eyes of each patient were examined at each visit and that patients on the average are seen twice a year, the mean frequency of visual field testing of both eyes is every fourth visit, i.e. approximately at 2 years interval. Corresponding interval for 2779 field tests are close to 3 years. With the same assumptions, fundus imaging was performed approximately every eighth to tenth year.

## Discussion

This was the first nationwide survey for benchmarking and to provide information about glaucoma management in Sweden. The high response rate, especially among public health care providers, allows us to draw some conclusions on the practices in Sweden.

This practice survey in combination with statistics and other available official information showed that glaucoma care constitutes a significant part of ophthalmology health care. More than a quarter of outpatient visits in ophthalmology health care were related to glaucoma. The management of glaucoma and suspect glaucoma thus constituted a considerable part of the workload of many ophthalmologists.

The study confirmed that providers of ophthalmic care in Sweden have access to adequate equipment for diagnosis and follow-up of patients with glaucoma. Computerized perimetry is generally available, and most ophthalmic health care providers also have access to cameras and/or image instruments for fundus evaluation. Similar access to equipment was found in a recent survey investigating glaucoma management in the United Kingdom (Gordon-Bennett et al. 2008), with the exception that digital imaging was more common in United Kingdom compared to Sweden. Thus, 66% of United Kingdom consultants had access to digital imaging instruments, whereas in Sweden, only 13% of the public departments and 3% of the private clinics were equipped with such an instrument.

The frequencies of visual field testing and fundus imaging were lower than expected. Our calculation is a best case scenario, using the highest estimate of number of field test. If the lower number of perimetries was correct, the corresponding intertest interval would be almost 3 years. Previous national guidelines (Heijl et al. 1998) recommended that patients with stable glaucoma should undergo visual field testing once or twice per year. More frequent examinations could be considered if faster progression was suspected. In contrast, the results of the present survey indicate considerably less frequent visual field examinations, on the average approximately only every 2 years. This is only half the recommended rate. The new Swedish glaucoma guidelines (Heijl et al. 2010) recommend 2–3 visual field tests each year for the first 2 years. This is based on calculations of the power of visual fields to detect an unacceptable rate of progression (Chauhan et al. 2008). Thus, modern principles of glaucoma care focus on the need to identify rapidly progressing patients early on in the disease, to prevent unnecessary damage and reduce IOP further.

In a retrospective chart review of newly diagnosed patients with glaucoma in the United States (Quigley et al. 2007), 83% had visual field examinations at 2-year interval or less. Thirty-six per cent of the subjects had a visual field test at least every year and 60% every 18 months. This indicates that the practice in the United States is more conformed to recommendations. However, the US study population only included newly diagnosed patients where the visual field examinations might be performed more often than in an unselected group of patients.

For fundus photography, a similar best case scenario calculation as for visual field rates indicates that on the average, optic nerve head documentation was performed only every 8–10 years. The very low frequency of fundus imaging shows that perimetry has not been replaced with imaging. A reason for the low rate of fundus imaging may well be that there is much less evidence-based support for the effectiveness of repeated fundus photographs in the follow-up of glaucoma (SBU 2008).

The low frequency of documentation of glaucoma damage with perimetry and imaging must clearly have influenced glaucoma management. Even if there are exceptions to the main rule of frequent perimetry (e.g. in very old patients or patients where IOP is already at the lowest attainable levels), an average frequency of testing visual fields every second year is unacceptably low. When damage is estimated at such infrequent intervals, estimation of rate of disease progression is largely impossible and cannot be achieved in less than at least 6 years (Chauhan et al. 2008). The large randomized glaucoma trials have shown that rates of disease progression are extremely variable among patients with glaucoma (Anderson et al. 2001; Heijl et al. 2009) and that progression is common also if the intraocular pressure is within the statistically normal limits (Leske et al. 2007). We are, therefore, forced to draw the conclusion that glaucoma care in Sweden seems to have largely been based on tonometry. This is not in line with the current thinking where assessment of damage and rate of progression are important factors to determine target pressure. It is noteworthy that the variations were large between geographical regions and health care providers. Most likely, some centres have followed earlier guidelines, but, if so, the standard of glaucoma care has been even lower in certain centres than the means reported here. University departments seemed to have an even lower frequency of perimetry than the total mean. This may in part be due to slightly different roles in glaucoma management where, e.g., postoperative visits and visits for second opinions are more frequent in university departments. Still, also among university departments, the variation was large. The highest frequency of perimetry was reported from Southern Region, where large-scale glaucoma studies and trials have been performed (Heijl & Bengtsson 2000; Grødum et al. 2002; Heijl et al. 2002) over the past decades. It is possible that the research interest has contributed to the higher frequency of visual field testing in this area.

Glaucoma care may be organized differently in different regions of the country. In this context, the South and Stockholm Regions seemed to represent extremes. In the Southern Region, 17% of all visits during the investigated week were related to glaucoma, while the corresponding figure for Stockholm was 34%. Similarly, the estimated numbers of glaucoma-related visits per 100 000 inhabitants, more than 70 years of age, were approximately 500 in the Southern Region and 850 in the Stockholm Region that week. It is therefore possible that patients with glaucoma were seen more frequently in Stockholm than in the South. If so, the difference in the frequency of visual field testing between regions may have been smaller.

Against the background of this practice survey and the conclusions following the literature review conducted within this project, it is clear that monitoring of glaucoma damage must improve in Sweden, particularly the number of visual field tests. Visual field testing should be more common at least during the first few years after diagnosis to allow relatively rapid detection of patients with high progression rates and assessment of the individual rate of progression (Chauhan et al. 2008; European Glaucoma Society 2008; Heijl et al. 2010). On the other hand, as glaucoma progression measured with global visual field indices often is linear (Bengtsson et al. 2009), it is clearly acceptable to measure glaucoma damage with long intervals (perhaps 2 years), if rate of progression has been measured over many years and is low enough not to threaten the patient’s quality of life during his/her remaining lifetime.

With the proof that IOP reduction strongly reduces the risk of disease progression to an extent where even a few mmHg of extra pressure reduction may reduce rate of progression in a clinically meaningful way (Gordon et al. 2002; Leske et al. 2003), knowledge of the intraocular pressure and the rate of progression becomes of paramount interest for effective, individualized glaucoma care.

As the cost of a visual field test is estimated to be only about 300 Swedish kronor (SEK) per examination, it should be possible to implement the suggested change with a modest amount of additional funding. Thus, an increase in the average frequency of visual field tests from one examination every 2 years to one every year would increase the cost of ophthalmology services by approximately 15 million SEK, corresponding to approximately 2% of the direct annual healthcare costs for glaucoma in Sweden. We hope that the new national glaucoma guidelines (Heijl et al. 2010) will explain and exert pressure so that Swedish glaucoma management will improve in this respect.

In conclusion, glaucoma care generated about a quarter of all patient visits in Swedish ophthalmic care. Access to diagnostic facilities was good. To meet modern standards of glaucoma care, glaucoma damage must be measured and followed more closely than at the time of the survey.
